# *Corynebacterium pseudotuberculosis* may be under anagenesis and biovar Equi forms biovar Ovis: a phylogenic inference from sequence and structural analysis

**DOI:** 10.1186/s12866-016-0717-4

**Published:** 2016-06-02

**Authors:** Alberto Oliveira, Pammella Teixeira, Marcela Azevedo, Syed Babar Jamal, Sandeep Tiwari, Sintia Almeida, Artur Silva, Debmalya Barh, Elaine Maria Seles Dorneles, Dionei Joaquim Haas, Marcos Bryan Heinemann, Preetam Ghosh, Andrey Pereira Lage, Henrique Figueiredo, Rafaela Salgado Ferreira, Vasco Azevedo

**Affiliations:** Departamento de Biologia Geral, Laboratório de Genética Celular e Molecular, Universidade Federal de Minas Gerais, Belo Horizonte, Minas Gerais Brazil; Departamento de Bioquímica e Imunologia, Laboratório de Genética Celular e Molecular, Universidade Federal de Minas Gerais, Belo Horizonte, Minas Gerais Brazil; Departmento de Genética, Universidade Federal do Pará, Pará, Brazil; Centre for Genomics and Applied Gene Technology, Institute of Integrative Omics and Applied Biotechnology (IIOAB), Nonakuri, Purba, Medinipur, WB-721172 India; Departamento de Medicina Veterinária Preventiva, Escola de Veterinária – Universidade Federal de Minas Gerais, Belo Horizonte, Minas Gerais Brazil; Departamento de Medicina Veterinária Preventiva e Saúde Animal, Faculdade de Medicina Veterinária e Zootecnia – Universidade de São Paulo, São Paulo, Brazil; Department of Computer Science, Virginia Commonwealth University, Richmond, VA USA; Aquacen, National Reference Laboratory for Aquatic Animal Diseases, Federal University of Minas Gerais, Belo Horizonte, Minas Gerais Brazil

**Keywords:** *Corynebacterium pseudotuberculosis*, Evolution, Molecular phylogeny, Structural biology

## Abstract

**Background:**

*Corynebacterium pseudotuberculosis* can be classified into two biovars or *biovars* based on their nitrate-reducing ability. Strains isolated from sheep and goats show negative nitrate reduction and are termed biovar Ovis, while strains from horse and cattle exhibit positive nitrate reduction and are called biovar Equi. However, molecular evidence has not been established so far to understand this difference, specifically if these *C. pseudotuberculosis* strains are under an evolutionary process.

**Results:**

The ERIC 1 + 2 Minimum-spanning tree from 367 strains of *C. pseudotuberculosis* showed that the great majority of biovar Ovis strains clustered together, but separately from biovar Equi strains that also clustered amongst themselves. Using evolutionarily conserved genes (*rpoB, gapA, fusA, and rsmE*) and their corresponding amino acid sequences, we analyzed the phylogenetic relationship among eighteen strains of *C. pseudotuberculosis* belonging to both biovars Ovis and Equi. Additionally, conserved point mutation based on structural variation analysis was also carried out to elucidate the genotype-phenotype correlations and speciation. We observed that the biovars are different at the molecular phylogenetic level and a probable anagenesis is occurring slowly within the species *C. pseudotuberculosis.*

**Conclusions:**

Taken together the results suggest that biovar Equi is forming the biovar Ovis. However, additional analyses using other genes and other bacterial strains are required to further support our anagenesis hypothesis in *C. pseudotuberculosis*.

**Electronic supplementary material:**

The online version of this article (doi:10.1186/s12866-016-0717-4) contains supplementary material, which is available to authorized users.

## Background

The genus *Corynebacterium* belongs to the bacterial phylum *Actinobacteria*, also known as *Actinomycetes*. This phylum comprises *Mycobacterium*, *Nocardia* and *Rhodococcus* genera, which together form a supra-generic group known by their initials as CMNR [1–3]. These organisms share some common features, such as:

(i) A specific well-organized cell wall mainly characterized by the presence of vast components of peptidoglycan, mycolic acid, and arabinogalactan [4–7];
(ii) high G + C content (47 %–74 %) [5];
(iii) Gram-positive [8].

Within the genus *Corynebacterium*, the species *Corynebacterium pseudotuberculosis* is reported to be a facultative intracellular pathogen in mammals [9, 10]. The pathologies associated with *C. pseudotuberculosis* are of great importance to veterinary medicine because this bacterium is considered the main etiologic agent of caseous lymphadenitis (CLA). CLA is characterized by abscess formation in the thorax and abdomen, or in major lymph nodes, causing dermonecrosis and finally resulting in hypertrophy in the affected region [9, 11]. CLA is mostly found in small ruminants (mainly sheep and goats), but other mammals, such as cattle, pigs, deer, sheep, horses, camels and even human beings, although with very rare incidences, can be affected [12, 13]. Curiously, infection by *C. pseudotuberculosis* may cause other diseases*,*such as ulcerative lymphangitis (UL), a pathology of lymphatic vessels of the lower extremities, particularly hind legs, which is most frequent in horses [14, 15].

In order to understand differences in clinical presentation by infection of *C. pseudotuberculosis*, some studies have proposed to classify this microorganism from genetic, morphological and biochemical points of view [16–18]. Especially based on its ability to breakdown nitrate [19, 20], *C. pseudotuberculosis* was classified into two biovars, Ovis and Equi. Strains isolated from sheep and goats, which are usually negative in nitrate reductase activity, were classified as biovar Ovis; whereas the strains isolated from horse and cattle, which are usually positive in the nitrate reduction test, were classified as biovar Equi.

Additionally, studies have attempted to define these two biovars (Ovis and Equi) using restriction endonucleases (*Eco*RV and *Pst*I) on chromosomal DNA or focusing on nitrate reduction determination methods [21]. Discrimination of both isolates was also possible using other methodologies, such as restriction fragment length polymorphism analysis of 16S ribosomal DNA [19, 20, 22] and pulsed-field gel electrophoresis (PFGE) in combination with biochemical analysis [23]. Other studies have investigated the possible evolutionary divergences of the genus *Corynebacterium* using 16S rRNA sequences [24–26], the preferred genetic tool used to characterize organisms taxonomically [27]. Although 16S rRNA (*rmsE*) gene sequencing is highly useful with regards to bacterial classification, published data has proven analysis of the partial nucleotide sequences of the RNA polymerase β-subunit gene (*rpoB*) is more accurate for *Corynebacterium* species [18, 28].

There is phenotypic evidence (nitrate test) and genotypic evidence (Enterobacterial repetitive intergenic consensus sequence-based - ERIC-PCR and Single-nucleotide polymorphism (SNP) analysis [29, 30]) showing differences between the two biovars. However, our goal in this paper is to investigate the evolutionary differences between biovars Equi and Ovis of *C. pseudotuberculosis*. Thus, in this study, we performed ERIC 1 + 2 PCR and evolutionary analysis, using maximum likelihood method in combination with gene and protein structural analysis, of genes *rsmE,* a vital component of the ribosome, and *rpoB*, a region of strong influence of RNA polymerase activity, to find a kinship and phylogenetic distances. To study the molecular divergences between biovar Ovis and Equi of *C. pseudotuberculosis,* we also considered other genes like *gapA*, which has been used as a target in taxonomic comparisons of bacteria, taking into account the functions that this gene infers in possible differences in the metabolism of carbohydrates, glycolysis and cell survival [31, 32], and *fusA*, that has been used for phylogenetic analysis and taxonomic classification of bacterial species of the genus *Pantoea*, a pathogen to humans and plants [33].

## Methods

### ERIC 1 + 2 PCR Minimum-spanning tree

The minimum-spanning tree (MST) was generated using ERIC1 + 2-PCR data from 367 *C. pseudotuberculosis* strains, including 226 biovar Ovis field strains [17, 29, 34], 139 biovar Equi field strains (34 strains with published data [29] and 105 strains isolated from equines in USA – unpublished data), type strain ATCC 19410^T^ and vaccine strain1002. The *C. pseudotuberculosis* ATCC 19410^T^ type strain and 1002 vaccine strain were genotyped by ERIC1 + 2-PCR one time in each of the four different assays gathered in the present study ([17, 29, 34] and unpublished data). The MST was built using UPGMA, to calculate the distance matrix, and Prim’s algorithm associated with the priority rule and permutation resampling [35, 36]. The MST presented is the top scoring tree, i.e., the tree with the highest overall reliability score.

### Dataset

Eighteen *C. pseudotuberculosis* strains with available genome sequences (Additional file 1) had their sequences of genes/proteins *rpoB, gapA, fusA, and rsmE* (Additional file 2) submitted to phylogenetic and structural analyses. ERIC1 + 2-PCR results were available for 13 of those 18 strains. All genome sequences were available from the NCBI database [37, 38]. Protein functional information was from UniProt database annotation [39].

### Alignment and phylogenetic analysis

We have used Clustal-X [40] for multiple sequence alignment (MSA) and Jalview [41] to visualize and edit the MSA**,** create phylogenetic trees, explore molecular structures and annotation. The analyses on the transition and transversion mutations were done using the software MEGA 6 [42]. Average Nucleotide Identity (ANI) [43] was employed to evaluate relatedness among strains in substitution of the labour-intensive DNA-DNA hybridization (DDH) technique.

In order to create the phylogenetic trees, we first obtained an evolutionary model adapted to the MSA. Therefore, we used Adaptive Server Evolution (http://www.datamonkey.org/) portal to define one evolutionary model. The outcome of these tests indicate the TN93 model [44]. Seaview [45] was then used to construct the tree based on the model previously presented by the Adaptive Server Evolution portal. The tree was created using the maximum likelihood method performed by PHYML [46], which is available in Seaview. Branch support consistencies were evaluated using the nonparametric bootstrap test [47] with 250 replicates and the approximate likelihood ratio test (ALRT) [48]. The viewing and editing of the tree was carried out using the Figtree tool [49, 50] that enabled us to either characterize different gene groups based on the bootstrap values calculation or to represent the evolutionary time scale. The multiple sequence alignments contain all four genes used in this work.

### Statistical analysis

The timetree was generated using the RelTime method [51]. Divergence times for all branching points in the user-supplied topology were calculated using the Maximum Likelihood method based on the General Time Reversible model [52]. Bars around each node represent 95 % confidence intervals which were computed using the method described by Tamura et al. [42]. The estimated log likelihood value of the topology shown is −34964.1082. Similar evolutionary rates were merged between ancestors and descendants so that many clocks were identified in the topology. The rate variation model allowed for some sites to be evolutionarily invariable ([+*I*], 29.1188 % sites). The tree is drawn to scale, with branch lengths proportional to the relative number of substitutions per site. Also, the statistic Tajima’s D [53] was used in order to compare the average number of pairwise differences with the number of segregating sites. The Tajima’s D statistical can be understood below:

$$ E\left[\pi \right] = \theta =E\left[\frac{S}{\varSigma \frac{n-1}{i=1}\frac{1}{i}}\right]=4N\upmu $$

Where *S* means the number of segregations sites, *n* the number of samples and *i* is the index of summations. Follows the Tajima’s D statistical test, there are factors that can change the expected values of *S* and *π* . The crux of Tajima’s D test statistic is the difference in the expectations for these two variables (which can be positive or negative). Finally, *D* is calculated by considering the differences between the two estimates of the population genetics parameter *θ*. The D value is obtained by dividing these differences, that is called *d* by the square root of its variance $\sqrt{\widehat{V}}(d)$.

$$ D = \frac{d}{\sqrt{\widehat{V}(d)}} $$

The alignment with all 4 genes from two *C. pesudotuberculosis* strains, vaccine strain 1002 from biovar Ovis and strain E19 from biovar Equi, and one *Arcanobacterium haemolyticum* strain, AH 20595 employed as an external group, were used for this statistical analysis. *P*-value less than 0.05 were used to reject the null hypothesis of equal rates between lineages.

### Estimation of the pattern of nucleotide substitution

In order to observe the probability of transition (G↔A) and transversion (A↔C) substitutions, the MSA, of all 4 genes from biovars Equi and Ovis, was taken into account, with the aim to determine the types of molecular changes that were occurring. The Maximum Composite Likelihood Estimate of the Pattern of Nucleotide Substitution method was used with the gamma model that corrects for multiple hits, taking into account the rate substitution differences between nucleotides and the inequality of nucleotide frequencies [54]. The analysis involved 19 (external group inside) nucleotide sequences. The transition/tranversion ratio (R), that is the number of these replacement, was calculated by *R* = [A*G*k_*1*_ + T*C*k_*2*_]/[(A + G)*(T + C)] with rate ratios of k_1_ evaluating purines and rate ratios of k_2_ evaluating pyrimidines. Codon positions included were 1st + 2nd + 3rd + Noncoding. All positions containing gaps and missing data were eliminated. There were a total of 6542 positions in the final dataset. Evolutionary analyses were conducted in MEGA6 [42].

### Structural analysis

For structural analysis, the gene sequences were translated to amino acid sequences using the Transeq program: http://www.ebi.ac.uk/Tools/emboss/. After the amino acid alignment, we constructed 3-D models of the proteins and interpreted the molecular differences and consequences. Comparative molecular modeling of proteins was performed with the software Modeller [55]. Additional file 3 presents the details of the template structures using information from NCBI [52] and PDB (Protein Data Bank) [56]. Twenty models were built for each of the templates using MODELLER and one model for each template. PyMOL V.1.5.0.4 was used to visualize three-dimensional protein structures (Schrödinger, LLC.). All homology models were evaluated using several different model evaluation tools such as PROCHECK [55], evaluating the stereochemistry quality, Discrete Optimized Protein Energy (DOPE) score [56], a statistical potential able to provide a energetic validation and RMSD obtained from a structural alignment with a protein with similar function, to provide an functional validation. The latter was carried out in the software PyMOL. For all analysis we selected amino acids that are closer than 4 angstroms (Å) from the variant amino acid.

*We want to inform that this work does not use any participants, children, parent or guardian.

## Results

### *C. pseudotuberculosis* biovar Ovis and biovar Equi strains clustered separately by ERIC 1 + 2 PCR

**Fig. 1:**
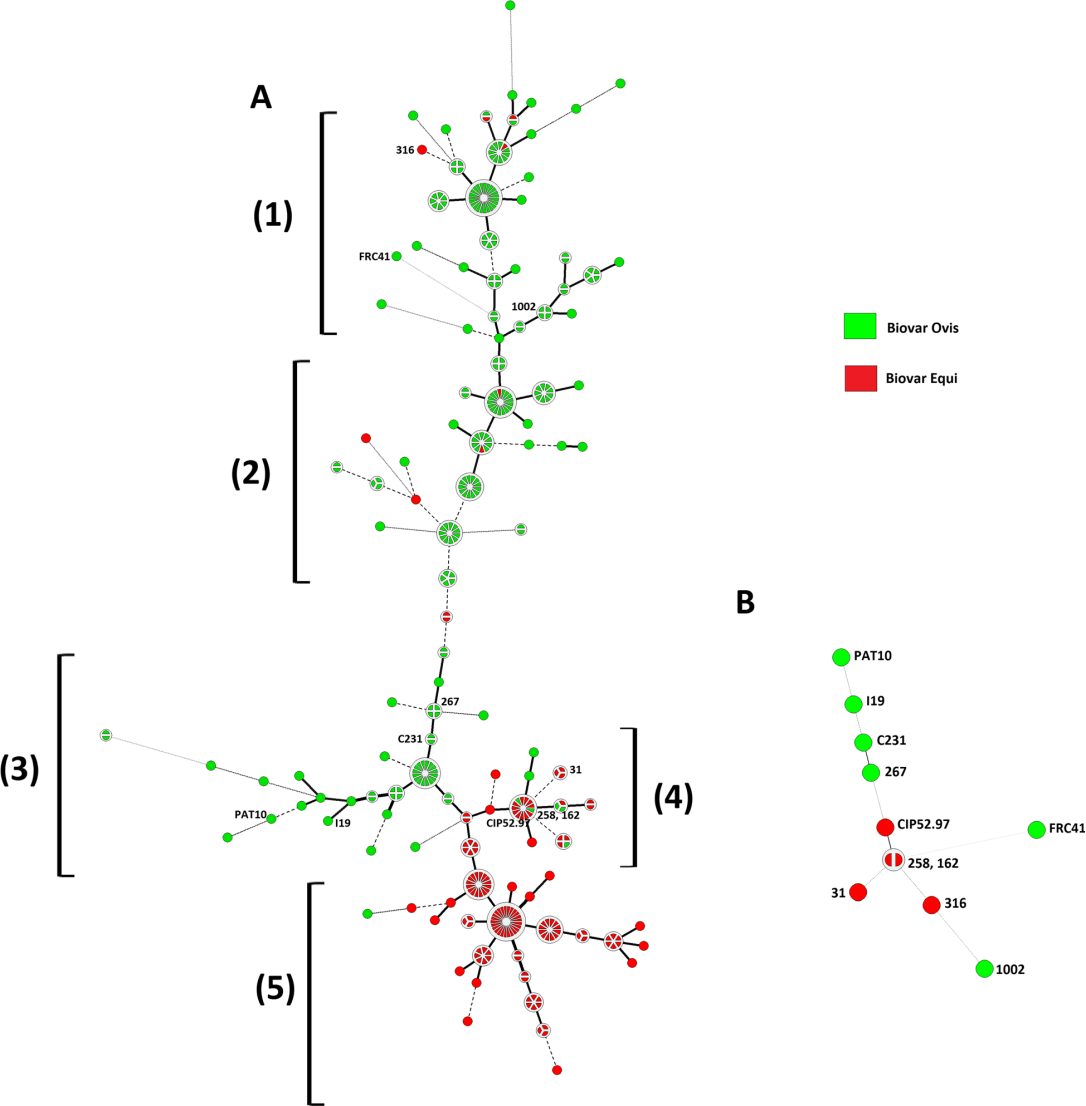
Minimal spanning tree by ERIC 1 + 2-PCR of 367 (**a**) and eleven (**b**) *C. pseudotuberculosis* strains. The branch length indicates the distance between the nodes as follows: () up to 1 %; () up to 5 %; () up to 10 %; () up to 15 %; and () above 15 %. The sizes of the nodes depend on the number of strains (their population size). Wedges in circles represent the proportion of *C. pseudotuberculosis* isolates from respective sources. The MST presented is the tree with the highest overall reliability score and was calculated using the UPGMA associated with the priority rule and permutation resampling using Bionumerics 7.1 (Applied Maths, Sint-Martens-Latem, Belgium)

The ERIC 1 + 2 Minimum-spanning tree from 367 strains (365 field strains, type strain ATCC 19410^T^ and vaccine strain 1002) of *C. pseudotuberculosis* showed that the great majority of biovar Ovis strains clustered together, but separately from biovar Equi strains that also clustered amongst themselves (Fig. 1-a) (Additional file 4). The same was observed when the minimum-spanning tree was constructed only from strains with complete genome sequences (Fig. 1-b) (Additional file 4). Moreover, analysis of all *C. pseudotuberculosis* strains depicted the existence of five major clonal complexes; three that clustered around biovar Ovis and two around biovar Equi strains.

The clonal complex labelled 1 was almost totally composed of *C. pseudotuberculosis* strains from Minas Gerais State, Brazil, whereas clonal complex 2 contained strains from the states of Minas Gerais, Pernambuco and São Paulo, Brazil. The last clonal complex related to *C. pseudotuberculosis* biovar Ovis strains (3) showed the most diverse composition, including strains from Argentina, Australia, Brazil (Minas Gerais, Bahia, and São Paulo), Egypt, Israel, Scotland and USA. *C. pseudotuberculosis* biovar Equiclonal complexes were mainly composed of strains from Egypt (4) and USA (5).

The repeatability observed for the *C. pseudotuberculosis* ATCC 19410^T^ and 1002 vaccine strain in ERIC1 + 2-PCR was 84 % considering the four different experiments conducted [17, 29, 34] and also the unpublished data from 105 strains.

### Statistical analysis and molecular phylogeny from *C. pseudotuberculosis* Ovis and Equi biovars

**Fig. 2:**
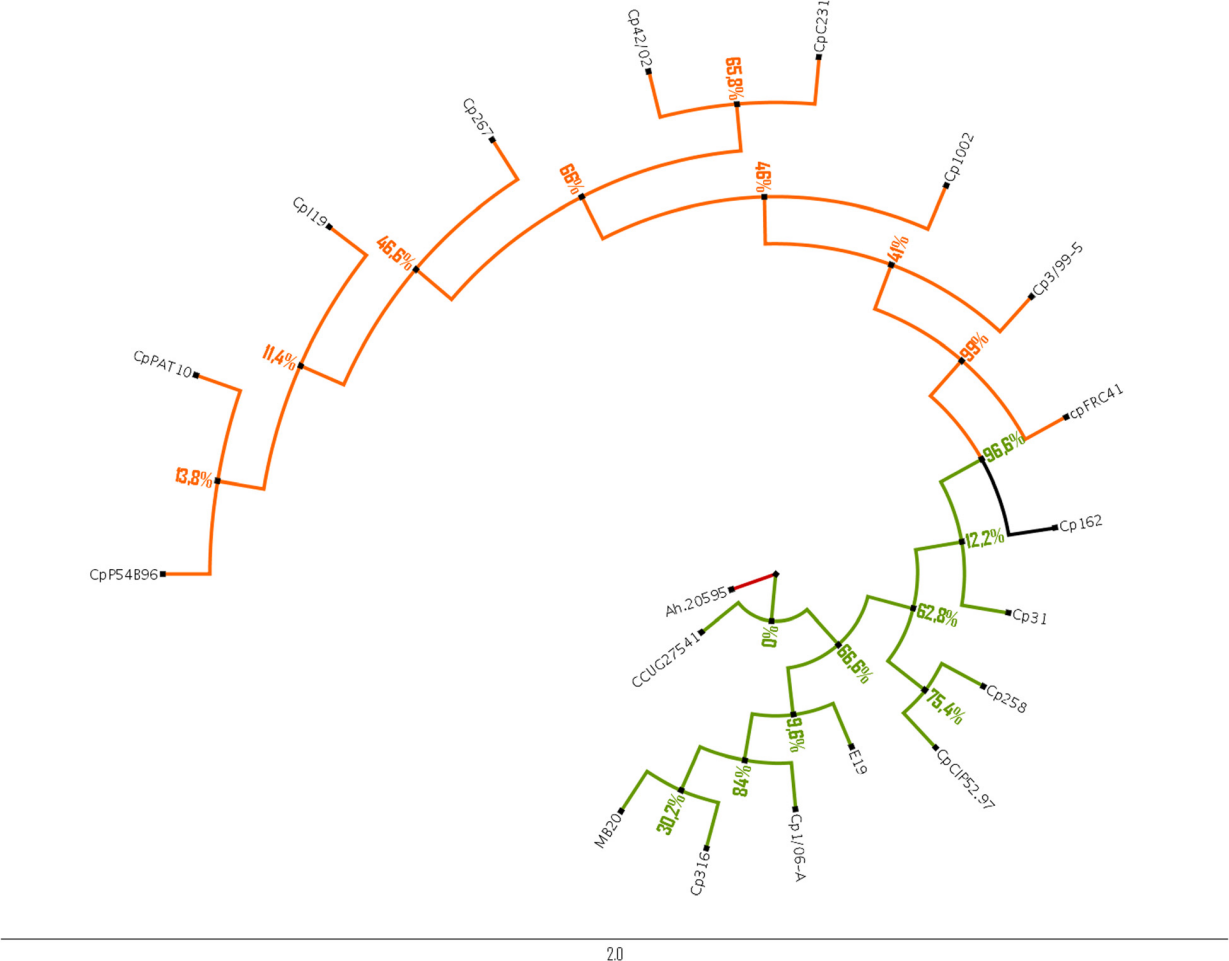
Phylogenetic tree of Equi and Ovis biovars determined by maximum likelihood method. Phylogenetic tree demonstrating the relationships of the *C. pseudotuberculosis* strains represented by biovars Ovis (orange) and Equi (green) showing their evolutionary differences. The tree is based on the results of distance matrix analyses of all 4 genes explored in this work. The topology of the tree was determined by performing maximum likelihood analyses. The outside group is highlighted in the brown edge. Boostrap values can be identified by the label on the left side and the nodes in the tree

The phylogenetic tree was carried out to understand the taxonomic distribution of biovars observed from ERIC-PCR. This result showed a small fragmentation of branches among the 18 *C. pseudotuberculosis* strains. For the analysis we added an external group using the gene sequences from *Arcanobacterium haemolyticum* (AH 20595) that are homologous to the four *C. pseudotuberculosis* genes analyzed. This can be visualized in the tree by a red edge (Fig. 2). The fragmented edges that belong to biovar Equi are highlighted in green and black (only strain 162), while the ones that belongs to biovar Ovis are highlighted in orange. The bootstrap value appears along the tree as well as in the legend. The result for ANI was 98 % (Additional file 5). The results of the timetree (Additional files 6 and 7) showed a difference among *C. pseudotuberculosis* strains of biovars Equi and Ovis, taking into account the number of genetic distances to a fraction of the time. Biovar Equi strains trees have different branch length and taxonomic separation. On the other hand, biovar Ovis strain trees present more similar genetic distances between them, without differences in branch lengths. In addition, the results obtained in Tajima’s D, shown in Table 1, point to the existence of differences among average numbers of pairwise differences with the number of segregating sites. These data can be supported in regards to a Value of Tajima’s D < 0, which was in our results −2.07.

**Table 1:** Results from Tajima’s neutrality test

*m*	*S*	*p*_s_	*Θ*	*π*	*D*
19	841	0.52	0.15	0.07	−2.07

The analysis involved 19 amino acid sequences. The coding data was translated assuming a genetic code table. All positions containing gaps and missing data were eliminated. There were a total of 1599 positions in the final dataset. Evolutionary analyses were conducted in MEGA6

*Abbreviations*: *m* = number of sequences, *n* = total number of sites, *S* = Number of segregating sites, *p*_s_ = *S*/*n*, *Θ* = *p*_s_/a_1_, *π* = nucleotide diversity, and *D* is the Tajima test statistic [52]

### Statistical differences and transition and transversion point mutations detected in the multiple sequence alignment from biovares Ovis and Equi

The statistical results of transitional and transversion replacement has pointed to the existence of specific points mutations for both biovars. For example, the Additional file 8 shows one of the arrows that for biovar Equi there is the presence of guanine instead of adenine, a nitrogenous base found only in biovar Ovis. Table 2 shows the distribution of the nucleotide variation in the complete alignment. We can observe that the percentage of transition mutations is higher than that of the transversion mutations. The nucleotide frequencies are 21.51 % (A), 22.95 % (T/U), 27.59 % (C), and 27.95 % (G). The transition/transversion rate ratios are *k*_*1*_ = 13.99 (purines) and *k*_*2*_ = 3.89 (pyrimidines). The overall transition/transversion bias is *R* = 4.35.

**Table 2:** Maximum composite likelihood estimate of the pattern of nucleotide substitution

	A	T	C	G
A	-	*2.11*	*2.53*	**35.92**
T	*1.98*	-	**9.86**	*2.57*
C	*1.98*	**8.2**	-	*2.57*
G	**27.65**	*2.11*	*2.53*	-

Each entry shows the probability of substitution (r) from one base (row) to another base (column) [54]. For simplicity, the sum of *r* values is made equal to 100. Rates of different transitional substitutions are shown in bold and those of transversionsal substitutions are shown in *italics*

### Specific amino acid variation among biovar Ovis and Equi

Next, we evaluated the location of these point mutations on protein structure and whether the physicochemical characteristics of the mutated residues differ between the two biovars. We considered the protein sequences from the four genes used in this study, and amino acid differences were evaluated based on a sequence alignment with proteins (Additional file 9) possessing the same function and known. After the alignment, the position of each mutation in the structure was analyzed in comparison to the active site of each protein. In general, mutations were located in the surface and distant from the active site region (Additional file 10).

For *fusA* protein observed two residue modifications (Val177Ile and Asp371Glu) when comparing the two biovars. In both cases the amino acids within each pair have the same physicochemical characteristics: both valine and isoleucine are nonpolar and hydrophobic, and aspartate and glutamate are both polar and negatively charged. Therefore, both mutations are conservative and the mutations most likely don’t affect the function of this protein (Additional file 11).

For *gapA* protein, we observed that Asparagine (Asn) was changed to Aspartic acid (Asp) at position 97 when in both the biovars*.* Both are polar amino acids, however they differ in physicochemical terms, since Asn is neutral and contains a hydrogen bond donor, while Asp is usually negatively charged at neutral pH. Additionaly, at position 207 another amino acid change was observed, from threonine (Thr) to isoleucin (Ile). In this case, the amino acids differ in size (Ile has a bigger side chain) and polarity (while Ile is nonpolar, Thr is a neutral polar amino acid). Interestingly, *C. pseudotuberculosis* strain 162 that belongs to biovar Equi does not show the same changes when compared to the other strains from the same biovar.

Comparative analysis of the 16S ribosomal RNA methyltransferase (*rsmE*) from *C. pseudotuberculosis* biovars Equi and Ovis showed a His18Arg variation at the N-terminal region and a Thr132Ala variation, at the C-terminal region. Both Histidine (His) and Arginine (Arg) located at position 18 have polar side chains and may be positively charged at neutral pH, however Arg has a much higher pKa. At position 132, the amino acids Threonine (Thr) and Alanine (Ala) differ functionally as Thr is a neutral polar amino acid and Ala is nonpolar (Additional file 10). The Table 3 shows all variations for these proteins.

**Table 3:** Distribution of amino acids from proteins in biovars. The amino acid variants positions present physicochemical differences, and are observed to be specific to a biovar type. Some variations exhibit an increase or decrease in the number of interactions between amino acids that influence the stability of the protein

Protein	Position	Amino acid	Biovar
*fusA*	177	Valine (V)	Equi
		Isoleucine (I)	Ovis
	371	Glutamine (E)	Equi
		Aspartic acid (D)	Ovis
*gapA*	97	Asparagine (N)	Equi
		Aspartic acid (D)	Ovis
	207	Threonine (T)	Equi
		Isoleucine (I)	Ovis
*rmsE*	18	Histidine (H)	Ovis
		Arginine (R)	Equi
	132	Threonine (T)	Ovis
		Alanine (A)	Equi

Finally, for the beta subunit of RNA polymerase (*rpoB*) only one residue difference, Ala979Thr, was observed when comparing biovars Equi and Ovis. In this case there is a change in polarity for these two amino acids (Additional file 10). Curiously *C. pseudotuberculosis* strains 1002, 3/99, and FRC41, belonging to biovar Ovis, shared the same amino acids when compared to biovar Equi strains (Table 4).

**Table 4:** Distribution of amino acids from protein *rpoB* in biovars. The position 979 has variation in amino acids: Alanine for some strains as 1002, 106, 3/99, 31, 258, 52.97, 162 and FRC41; while the same position is Threonine for other biovars as 42/02, 267, 231, I19, P54B96 and PAT10

Alanine (A)^979^		Threonine (T)^979^	
Strain	Biovar	Strain	Biovar
1002	Ovis	42/02	Ovis
106	Equi	267	
3/99	Ovis	231	
31	Equi	I19	
258	Equi	P54B96	
52.97	Equi	PAT10	
162	Equi		
FRC41	Ovis		

## Discussion

We earlier suggested from data of 102 *C. pseudotuberculosis* strains that *C. pseudotuberculosis* biovar Ovis and Equi exhibited different ERIC1 + 2-PCR clustering pattern (Dorneles et al., [29]). The present data from 373 strains also showed a clear difference in clustering pattern between *C. pseudotuberculosis* biovar Ovis and Equi, with a few exceptions (Fig. 1-a). However, as the present MST results were based on a representative number of genotyped strains (139 *C. pseudotuberculosis* biovars Equi field strains, 226 *C. pseudotuberculosis* biovar Ovis field strains, from twelve countries and isolated from eight different hosts), it allows us to make more reliable inferences. The five largest clonal complexes were observed in the MST, three were mainly related to *C. pseudotuberculosis* biovar Ovis strains (1, 2, and 3) and two with *C. pseudotuberculosis* biovar Equi strains (4 and 5), from which other clonally related isolated groups emerge. The distinct clustering pattern of *C. pseudotuberculosis* biovar Ovis and Equi strains might reflect the number of genes specific to each biovar [57].

In order to understand the clustering formation, we have used algorithms and techniques to construct qualitative phylogenetic trees to explain the best possible evolutionary relationship between biovars Ovis and Equi from *C. pseudotuberculosis* strains. The difference in the biovars can be clearly observed from the ERIC 1 + 2-PCR MST and phylogenetic tree (Figs. 1 and 2). However, it was not possible to determine if the two biovars belongs to different species based on the results obtained after the phylogenetic tree and average nucleotide identity (ANI) analyses. Although there are some genetic and biochemical differences between them, it was not clear whether the branches, observed at phylogenetic tree, have distinct groups of organisms. Based on our data, we hypothesized that biological speciation may be occurring, for instance, anagenesis, which is a process of progressive evolution of species involving changes in the gene frequency of a population [58].

Based on the anagenesis mode of speciation, we have hypothesized that speciation is occurring within the *C. pseudotuberculosis* at a molecular level. The genes *rmsE*, *rpoB*, *fusA*, and *gapA* that we used are strong candidates for phylogenetic analysis because they are involved in many important cellular processes, such as maintenance of cellular integrity, cell survival and several metabolic reactions [31–33]. According to Dorela et al*.* [8], *rpoB* is a relevant gene used to explore evolutionary routes serving as a tool to search for new species, differently from 16S rRNA gene sequencing data, that presents resolution problems at genus and/or species level. In our study, it was observed that the 16S sequence may be better in characterizing the molecular differences (mainly in structural analysis) between the two biovars, when compared to the *rpoB* gene. Our multiple sequence alignments analysis showed that all the genes have transition and transversion point mutations, which is defining and separating *C. pseudotuberculosis* biovar Equi from Ovis*.* In addition, it was observed from the timetree analysis that differences between the two *C. pseudotuberculosis* biovars were due to time of acquired mutations. It is possible to observe that the strains of biovar Equi are more prone to have mutations while those of *C. pseudotuberculosis* biovar Ovis were more stable. It is not possible, even with these findings, to state that the two groups constitute different species. However, taking into account the time slice versus genetic differences, *C. pseudotuberculosis* strains belonging to biovar Equi are under a constant mutational process, regarding the lengths of the branches. Considering the analyses of *C. pseudotuberculosis* strain 162, that have phylogenetic features approaching biovar Ovis, we interpret that some changes are being stabilized giving rise to strains of biovar Ovis. This event may indicate that a biological speciation may be occurring slowly in *C. pseudotuberculosis*. Our hypothesis is that an anagenesis process is happening from *C. pseudotuberculosis* biovar Equi to *C. pseudotuberculosis* biovar Ovis. Such anagenesis is observed when a sufficient number of mutations are fixed in a population, which makes the emergence of a new phenotype possible in the future. Based on our data, we dismissed the possibility of a cladogenesis event because we did not observe the transformation of an organism into two others, but rather the possible genesis of one organism into another. Therefore, it is highly likely that the mutations described in this study strengthen the idea of anagenesis.

Also, the results from Tajima’s D statistical test give us the biological interpretation that the proportion of mutations that alter codons for amino acids is higher than expected in both biovars and the population is evolving as per mutation-drift equilibrium. Furthermore, also is possible be interpreted rare alleles present at low frequencies in both biovars and population expansion after a recent bottleneck. Our structural analysis showed that none of the described mutations occurred within these sites or in positions previously described as critical for activity. Therefore, probably they do not affect protein function, but they are useful to indicate phylogenetic distance. For example, it was observed that *C. pseudotuberculosis* strain 162, which belongs to biovar Equi, is phylogenetically closer to biovar Ovis strains than to biovar Equi strains, as observed in the phylogenetic tree (Fig. 2). Probably this phylogenetic rapprochement is due to *C. pseudotuberculosis* strain 162 present Ovis specific mutations, although it is included in Equi biovar based on the nitrate test. Regarding this observation we hypothesize that biovar Ovis is being originated from biovar Equi, as mentioned before, as *C. pseudotuberculosis* 162 strain shares point mutations from both biovars.

Taking into account the reports discussed in the literature, many differences within the genomes of some *C. pseudotuberculosis* strains were reported [8, 29, 57]. Recently, Almeida et al. [30] (unpublished data) found a 99 % concordance in detection by PCR of gene *narG*, responsible for nitrate reduction, and nitrate reduction test, suggesting that *C. pseudotuberculosis* biovars Equi could be identified by the presence of gene *narG*, whereas biovar Ovis does not present it. Guimarães et al. [34] showed a typing method based on PCR (ERIC-PCR), which proved to be a good method to discriminate genetic differences among *C. pseudotuberculosis* strains. Using this method it was possible to observe that *C. pseudotuberculosis* biovar Ovis and biovar Equi strains clustered separately [29]. The differences in the clustering pattern of *C. pseudotuberculosis* biovar Ovis and biovar Equi strains could reflect the number of specific genes in each biovar [57]. This fact is evident from the complete genome analyses of 18 *C. pseudotuberculosis* strains; among the total 1504 genes, it was shown that *C. pseudotuberculosis* biovar Ovis contains 314 orthologous genes that are shared by all strains from this biovar but are absent from one or more strains of *C. pseudotuberculosis* biovar Equi [57]. Furthermore, *C. pseudotuberculosis* biovar Equi strains have 95 core genes that are absent from one or more strains of *C. pseudotuberculosis* biovar Ovis [57]*.* Soares et al. [57], working with pathogenicity islands (PAI), found differences in some genes related to *pilus* formation in *C. pseudotuberculosis* biovar Equi and biovar Ovis strains. *Pilus* gene clusters are acquired normally in block through horizontal gene transfer and are composed of a specific sortase gene and the major, base and tip pilin genes. Genetic variation was found in genome analyses between the two *C. pseudotuberculosis* biovars (Almeida et al., [30]). *C. pseudotuberculosis* biovar Ovis strain VD57 had its genome analysed for the presence of SNP’s, and the average variation found when it was compared to *C. pseudotuberculosis* biovar Ovis strains was 823 nucleotides, whereas when compared to the *C. pseudotuberculosis* biovar Equi strains that number increased to 25285.3 SNP’s.

Regarding the anagenesis theory, we suggest that *C. pseudotuberculosis* biovar Equi is forming, after some genomic changes, *C. pseudotuberculosis* biovar Ovis. Hence, we postulated that *C. pseudotuberculosis* biovar Equi strains could be evolutionarily older compared to *C. pseudotuberculosis* biovar Ovis strains.

## Conclusions

*C. pseudotuberculosis* biovar Equi and biovar Ovis contain different molecular characteristics, although they belong to the same species. The formation of a new species is an event that happens very slowly in nature. It is possible to interpret that a speciation process is occurring from the anagenesis event. With regard to the statistical data, analysis of sequence and structure of proteins addressed in this study, we conclude that *C. pseudotuberculosis* biovar Ovis is being formed from *C. pseudotuberculosis* biovar Equi through anagenesis.

## Abbreviations

CLA, caseous lymphadenitis; DOPE, discrete optimized protein energy; ERIC-PCR, enterobacterial repetitive intergenic consensus sequence-based; MSA, multiple sequence alignment; MST, minimum-spanning tree; NCBI, National Center Biotechnology Institute; PDB, Protein Data Bank; PFGE, pulsed-field gel electrophoresis; UL, ulcerative lymphangitis; Uniprot, Universal Protein Resource.

## Additional files

Additional file 1: Information about the strains of *C. pseudotuberculosis* in this work. In total, 18 strains were used of which nine strains were Equi and nine strains were Ovis as tabulated below. (PDF 19 kb) Additional file 2: Information about the genes of *C. pseudotuberculosis*. All four genes were used in this work. These were united in the same alignment for phylogenetic analysis. (PDF 7 kb) Additional file 3: Ratio templates used for molecular modeling. € Resolution of the structure was determined by experimental methods of electron microscopy. (PDF 35 kb) Additional file 4: Bionumerics 7.1 (Applied Maths, Sint-Martens-Latem, Belgium) (BMP 1582 kb) Additional file 5: Average Nucleotide Identity (ANI) from biovar ovis and biovar equi. Typically, the ANI values between genomes of the same species are above 95 %. Our results show the value 98.76 %, which we expected due to the results of phylogeny. One-way ANI 1: 98.64 % (SD: 1.15 %), from 67439 fragments. One-way ANI 2: 98.69 % (SD: 1.20 %), from 101821 fragments. Two-way ANI: 98.76 % (SD: 0.92 %), from 34975 fragments. (PNG 435 kb) Additional file 6: Molecular clock analysis by Maximum Likelihood method. Relationship between molecular divergence and time. The analysis involved 19 amino acid sequences. All positions containing gaps and missing data were eliminated. There were a total of 6514 positions in the final dataset. Evolutionary analyses were conducted in MEGA6. (PDF 55 kb) Additional file 7: Results from comparative test with molecular clocks using the Maximum Likelihood method and without molecular clock of strains. (PDF 8 kb) Additional file 8: Fragment of multiple sequence alignment. The figure shows transition (arrows in blue) and transversion (red arrow) point mutations observed in nucleotide sequences from both biovars Equi and Ovis. (TIF 164 kb) Additional file 9: Fragment of multiple sequence alignment of protein. The figure shows the substitutions of amino acids, in which most of the cases the changes modify the physicochemical characteristics. (TIF 216 kb) Additional file 10: Representation of the structure of *gapA, rsmE* and *rpoB* by molecular modeling. The molecular differences between the amino acid from biovars Equi and Ovis induce an increase in the number of chemical bonds between amino acids that are close to the variant residue. (TIF 4028 kb) Additional file 11: Structure of the *fusA* protein in the biovars Equi and Ovis analyzed by molecular modeling [55]. Expansion of 4 angstroms (Å) from the variant amino acid where it is possible to identify clusters of neighboring residues interacting among themselves. (A) Variation between V ↔ I, (B) Variation between D ↔ E. (TIF 2499 kb)

## References

[1] Hard GC (1969). Corynebacterium ovis Electron Microscopic Examination of Corynebacterium ovis. J Bacteriol.

[2] Paule BJ A, Meyer R, Moura Costa LF, Bahia RC, Carminati R, Regis LF, Vale VLC, Freire SM, Nascimento I, Schaer R, Azevedo V (2004). Three-phase partitioning as an efficient method for extraction/concentration of immunoreactive excreted-secreted proteins of Corynebacterium pseudotuberculosis. Protein Expr Purif.

[3] Songer JG (1997). Bacterial phospholipases and their role in virulence. Trends Microbiol.

[4] Bayan N, Houssin C, Chami M, Leblon G (2003). Mycomembrane and S-layer: two important structures of Corynebacterium glutamicum cell envelope with promising biotechnology applications. J Biotechnol.

[5] Funke G, Lawson PA, Collins MD (1995). Heterogeneity within human-derived centers for disease control and prevention (CDC) coryneform group ANF-1-like bacteria and description of Corynebacterium auris sp. nov. Int J Syst Bacteriol.

[6] Hall V (2003). Corynebacterium atypicum sp. nov., from a human clinical source, does not contain corynomycolic acids. Int J Syst Evol Microbiol.

[7] Hard GC (1975). Comparative toxic effect of the surface lipid of Corynebacterium ovis on peritoneal macrophages. Infect Immun.

[8] Dorella FAD, Gustavo L, Achecoa CP, Liveirab SCO, Iyoshia AM, Zevedoa VA. Corynebacterium pseudotuberculosis: microbiology, biochemical properties, pathogenesis and molecular studies of virulence. Vet Res. 2006;37:201–218 10.1051/vetres:2005056 16472520

[9] Williamson LH (2001). Caseous lymphadenitis in small ruminants. Vet Clin North Am Food Anim Pract.

[10] Trost E, Ott L, Schneider J, Schröder J, Jaenicke S, Goesmann A, Husemann P, Stoye J, Dorella FA, Rocha FS, Soares SDC, D’Afonseca V, Miyoshi A, Ruiz J, Silva A, Azevedo V, Burkovski A, Guiso N, Join-Lambert OF, Kayal S, Tauch A (2010). The complete genome sequence of Corynebacterium pseudotuberculosis FRC41 isolated from a 12-year-old girl with necrotizing lymphadenitis reveals insights into gene-regulatory networks contributing to virulence. BMC Genomics.

[11] Dorella FA, Pacheco LG, Seyffert N, Portela RW, Meyer R, Miyoshi A, Azevedo V (2009). Antigens of Corynebacterium pseudotuberculosis and prospects for vaccine development. Expert Rev Vaccines.

[12] Marchand CH, Salmeron C, Bou Raad R, Méniche X, Chami M, Masi M, Blanot D, Daffé M, Tropis M, Huc E, Maréchal P, Decottignies P, Bayan N. Biochemical disclosure of the mycolate outer membrane of Corynebacterium glutamicum. J Bacteriol. 2012;194:587–97. 10.1128/JB.06138-11 PMC3264076 22123248

[13] Brown CC, Olander HJ, Alves SF (1987). Synergistic hemolysis-inhibition titers associated with caseous lymphadenitis in a slaughterhouse survey of goats and sheep in Northeastern Brazil. Can J Vet Res.

[14] Doherr MG, Carpenter TE, Wilson WD, Gardner IA (1998). Application and evaluation of a mailed questionnaire for an epidemiologic study of Corynebacterium pseudotuberculosis infection in horses. Prev Vet Med.

[15] Britz E, Spier SJ, Kass PH, Edman JM, Foley JE (2014). The relationship between Corynebacterium pseudotuberculosis biovar equi phenotype with location and extent of lesions in horses. Vet J.

[16] Judson R, Songer JG (1991). Corynebacterium pseudotuberculosis: in vitro susceptibility to 39 antimicrobial agents. Vet Microbiol.

[17] Dorneles EMS, Santana JA, Andrade GI, Santos ELS, Guimaraes AS, Mota RA, Santos AS, Miyoshi A, Azevedo V, Gouveia AMG, Lage AP, Heinemann MB (2012). Molecular characterization of Corynebacterium pseudotuberculosis isolated from goats using ERIC-PCR. Genet Mol Res.

[18] Khamis A, Raoult D, Scola BLA (2005). Comparison between rpoB and 16S rRNA Gene Sequencing for Molecular Identification of 168 Clinical Isolates of Corynebacterium Comparison between rpoB and 16S rRNA Gene Sequencing for Molecular Identification of 168 Clinical Isolates of Corynebacterium. J Clin Microbiol.

[19] Costa LR, Spier SJ, Hirsh DC (1998). Comparative molecular characterization of Corynebacterium pseudotuberculosis of different origin. Vet Microbiol.

[20] Sutherland SS, Hart RA, Buller NB (1996). Genetic differences between nitrate-negative and nitrate-positive C. pseudotuberculosis strains using restriction fragment length polymorphisms. Vet Microbiol.

[21] Songer JG, Beckenbach K, Marshall MM, Olson GB, Kelley L (1988). Biochemical and genetic characterization of Corynebacterium pseudotuberculosis. Am J Vet Res.

[22] Vaneechoutte M, Riegel P, de Briel D, Monteil H, Verschraegen G, De Rouck A, Claeys G (1995). Evaluation of the applicability of amplified rDNA-restriction analysis (ARDRA) to identification of species of the genus Corynebacterium. Res Microbiol.

[23] Connor KM, Quirie MM, Baird G, Donachie W (2000). Characterization of United Kingdom Isolates of Corynebacterium pseudotuberculosis Using Pulsed-Field Gel Electrophoresis Characterization of United Kingdom Isolates of Corynebacterium pseudotuberculosis Using Pulsed-Field Gel Electrophoresis. J Clin Microbiol.

[24] Khamis A, Raoult D, La Scola B (2005). Comparison between rpoB and 16S rRNA gene sequencing for molecular identification of 168 clinical isolates of Corynebacterium. J Clin Microbiol.

[25] Bujnicki JM (2000). Phylogenomic analysis of 16S rRNA:(guanine-N2) methyltransferases suggests new family members and reveals highly conserved motifs and a domain structure similar to other nucleic acid amino-methyltransferases. FASEB J.

[26] Pascual C, Lawson PA, Farrow JA, Gimenez MN, Collins MD (1995). Phylogenetic analysis of the genus Corynebacterium based on 16S rRNA gene sequences. Int J Syst Bacteriol.

[27] Balch WE, Fox GE, Magrum LJ, Woese CR, Wolfe RS (1979). Methanogens: reevaluation of a unique biological group. Microbiol Rev.

[28] Khamis A, Raoult D, La Scola B (2004). rpoB gene sequencing for identification of Corynebacterium species. J Clin Microbiol.

[29] Dorneles EMS, Santana JA, Ribeiro D, Dorella FA, Guimaraes AS, Moawad MS, Selim SA, Garaldi ALM, Miyoshi A, Ribeiro MG, Gouveia AMG, Azevedo V, Heinemann MB, Lage AP (2014). Evaluation of ERIC-PCR as Genotyping Method for Corynebacterium pseudotuberculosis Isolates. PLoS One.

[30] Almeida, s., Sandeep Tiwari, Mariano, d., rocha, f. s., Jamal, Syed Babar, Coimbra, n. a. r., Raittz, r. t., Dorella, f. a., Carvalho, a. f., Pereira, f. l., Leal, c. a. g., Debmalya Barh, Ghosh, p., Figueiredo, h. c. p., Moura-Costa, l. f., Portela, r. w V: The Genome Anatomy of Corynebacterium pseudotuberculosis VD57 a Highly Virulent Strain Causing Caseous lymphadenitis. Stand Genomic Sci 2015;57:1-8. 10.1186/s40793-016-0149-7 PMC4826502 27066196

[31] Toyoda K, Teramoto H, Inui M, Yukawa H (2009). Involvement of the LuxR-type transcriptional regulator RamA in regulation of expression of the gapA gene, encoding glyceraldehyde-3-phosphate dehydrogenase of Corynebacterium glutamicum. J Bacteriol.

[32] Toyoda K, Teramoto H, Inui M, Yukawa H (2008). Expression of the gapA gene encoding glyceraldehyde-3-phosphate dehydrogenase of Corynebacterium glutamicum is regulated by the global regulator SugR. Appl Microbiol Biotechnol.

[33] Delétoile A, Decré D, Courant S, Passet V, Audo J, Grimont P, Arlet G, Brisse S (2009). Phylogeny and identification of Pantoea species and typing of Pantoea agglomerans strains by multilocus gene sequencing. J Clin Microbiol.

[34] Guimarães ADS, Dorneles EMS, Andrade GI, Lage AP, Miyoshi A, Azevedo V, Gouveia AMG, Heinemann MB (2011). Molecular characterization of Corynebacterium pseudotuberculosis isolates using ERIC-PCR. Vet Microbiol.

[35] Feil EJ, Li BC, Aanensen DM, William P, Spratt BG, Hanage WP (2004). eBURST : Inferring Patterns of Evolutionary Descent among Clusters of Related Bacterial Genotypes from Multilocus Sequence Typing Data eBURST : Inferring Patterns of Evolutionary Descent among Clusters of Related Bacterial Genotypes from Multilocus Sequen. J Bacteriol.

[36] Salipante SJ, Hall BG (2011). Inadequacies of minimum spanning trees in molecular epidemiology. J Clin Microbiol.

[37] Sayers EW, Barrett T, Benson DA, Bryant SH, Canese K, Chetvernin V, Church DM, DiCuccio M, Edgar R, Federhen S, Feolo M, Geer LY, Helmberg W, Kapustin Y, Landsman D, Lipman DJ, Madden TL, Maglott DR, Miller V, Mizrachi I, Ostell J, Pruitt KD, Schuler JD, Sequeira E, Sherry ST, Shumway M, Sirotkin K, Souvorov A, Starchenko G, Tatusova TA. Database resources of the National Center for Biotechnology Information. Nucleic Acids Res. 2009;37(Database issue):D5–D15. 10.1093/nar/gkn741 PMC2686545 18940862

[38] Benson DA, Karsch Mizrachi I, Lipman DJ, Ostell J, Sayers EW (2009). GenBank. Nucleic Acids Res.

[39] Apweiler R, Bateman A, Martin MJ, O’Donovan C, Magrane M, Alam-Faruque Y, Alpi E, Antunes R, Arganiska J, Casanova EB, Bely B, Bingley M, Bonilla C, Britto R, Bursteinas B, Chan WM, Chavali G, Cibrian-Uhalte E, Silva A, Giorgi M, Fazzini F, Gane P, Castro LG, Garmiri P, Hatton-Ellis E, Hieta R, Huntley R, Legge D, Liu W, Luo J. Activities at the Universal Protein Resource (UniProt). Nucleic Acids Res. 2014;42:D191–8. 10.1093/nar/gkt1140 PMC3965022 24253303

[40] Larkin MA, Blackshields G, Brown NP, Chenna R, McGettigan PA, McWilliam H, Valentin F, Wallace IM, Wilm A, Lopez R, Thompson JD, Gibson TJ, Higgins DG (2007). Clustal W and Clustal X version 2.0.. Bioinformatics.

[41] Waterhouse AM, Procter JB, Martin DM A, Clamp M, Barton GJ (2009). Jalview Version 2--a multiple sequence alignment editor and analysis workbench. Bioinformatics.

[42] Tamura K, Stecher G, Peterson D, Filipski A, Kumar S (2013). MEGA6: Molecular Evolutionary Genetics Analysis version 6.0.. Mol Biol Evol.

[43] Goris J, Konstantinidis KT, Klappenbach JA, Coenye T, Vandamme PTJ (2007). DNA-DNA hybridization values and their relationship to whole-genome sequence similarities. Int J Syst Evol Microbiol.

[44] Tamura K, Nei M (1993). Estimation of the number of nucleotide substitutions in the control region of mitochondrial DNA in humans and chimpanzees. Mol Biol Evol.

[45] Gouy M, Guindon S, Gascuel O (2010). SeaView version 4: A multiplatform graphical user interface for sequence alignment and phylogenetic tree building. Mol Biol Evol.

[46] Guindon S, Gascuel O (2003). A Simple, Fast, and Accurate Algorithm to Estimate Large Phylogenies by Maximum Likelihood. Syst Biol.

[47] Felsenstein J (1985). Confidence Limits on Phylogenies: An Approach Using the Bootstrap. Evolution (N Y).

[48] Anisimova M, Gascuel O (2006). Approximate likelihood-ratio test for branches: A fast, accurate, and powerful alternative. Syst Biol.

[49] Suchard MA, Rambaut A (2009). Many-Core Algorithms for Statistical Phylogenetics. Bioinformatics.

[50] Pybus OG, Rambaut A, Harvey PH (2000). An integrated framework for the inference of viral population history from reconstructed genealogies. Genetics.

[51] Tamura K, Battistuzzi FU, Billing-Ross P, Murillo O, Filipski A, Kumar S (2012). Estimating divergence times in large molecular phylogenies. Proc Natl Acad Sci U S A.

[52] Nei M, Kumar S, Nei M, Kumar S (2000). Molecular Evolution and Phylogenetics.

[53] Tajima F (1989). Statistical Method for Testing the Neutral Mutation Hypothesis by DNA Polymorphism. Genetics.

[54] Tamura K, Nei M, Kumar S (2004). Prospects for inferring very large phylogenies by using the neighbor-joining method. Proc Natl Acad Sci U S A.

[55] Eswar N, Webb B, Marti-Renom MA, et al. Comparative protein structure modeling using MODELLER. Curr Protoc Protein Sci. 2007;Chapter 2:Unit 2.9. doi:10.1002/0471140864.ps0209s50. 10.1002/0471140864.ps0209s50 18429317

[56] Bernstein FC, Koetzle TF, Williams GJ, Meyer EE Jr., Brice MD, Rodgers JR, Kennard O, Shimanouchi T, Tasumi M. The Protein Data Bank: A Computer-based Archival File For Macromolecular Structures. J of Mol Biol. 1977;112(535). 10.1016/s0022-2836(77)80200-3 875032

[57] Soares SC, Silva A, Trost E, Blom J, Ramos R, Carneiro A, Ali A, Santos AR, Pinto AC, Diniz C, Barbosa EG V, Dorella FA, Aburjaile S, Rocha FS, Nascimento KKF, Guimaraes LC, Almeida S, Hassan SS, Bakhtiar SM, Pereira UP, Abreu VAC, Schneider MPC, Miyoshi A, Tauch A, Azevedo V. The pan-genome of the animal pathogen Corynebacterium pseudotuberculosis reveals differences in genome plasticity between the biovar ovis and equi strains. PLoS One. 2013;8:e53818. 10.1371/journal.pone.0053818 PMC3544762 23342011

[58] Sons JW (1978). The hypercycle: A principle of natural self-organization. Am J Vet Res.

