# Supplementary material for: Corynebacterium pseudotuberculosis may be under anagenesis and biovar Equi forms biovar Ovis: a phylogenic inference from sequence and structural analysis
Source: BMC Microbiol. 2016 Jun 2;16:100. doi: 10.1186/s12866-016-0717-4 (PMC4890528; doi:10.1186/s12866-016-0717-4)
Supplement: Additional file 1: — Information about the strains of C. pseudotuberculosis in this work. In total, 18 strains were used of which nine strains were Equi and nine strains were Ovis as tabulated below. (PDF 19 kb) [file 12866_2016_717_MOESM1_ESM.pdf]

Additional file 1. Information about the strains of *C. pseudotuberculosis* in this work. In total, 18 strains were used of which nine strains were *equi* and nine strains were *ovis* as tabulated below.

Designation	Animal/Host	Biovar	Country of Isolation	Year of isolation	NCBI access
**P54B96**	Antelope	Ovis	South Africa	2009	CP003385
**I19**	Bovine	Ovis	Israel	Not informed	CP002251
**31**	Buffalo	Equi	Egypt	Not informed	BioProject ID 73223
**162**	Camel	Equi	UK	1999	BioProject ID 89445
**1002**	Goat	Ovis	Brazil	1971	CP001809
**258**	Horse	Equi	Belgium	Not informed	CP003540.2
**CIP52.97**	Horse	Equi	Kenya	1952	CP003061
**316**	Horse	Equi	US California	2010	CP003077
**1/06 A**	Horse	Equi	USA California	Not informed	CP003082
**FRC41**	Human	Ovis	France	2006	CP002097

**267**	Lhama	Ovis	US California	2008	BioProject ID 73515
**C231**	Sheep	Ovis	Australia	1983	CP001829
**PAT10**	Sheep	Ovis	Argentina	2007	CP002924
**42/02-A**	Sheep	Ovis	Australia	Not informed	CP003062
**3/99-5**	Sheep	Ovis	Scotland	Not informed	CP003152
**CCUG27541**	Human	Equi	Oslo	2014	JPJB00000000.1
**MB20**	Horse	Equi	Vacaville USA	2014	JPUV00000000.1
**E19**		Equi	Chile	Not informed	CP012136.1
