# Supplementary material for: Corynebacterium pseudotuberculosis may be under anagenesis and biovar Equi forms biovar Ovis: a phylogenic inference from sequence and structural analysis
Source: BMC Microbiol. 2016 Jun 2;16:100. doi: 10.1186/s12866-016-0717-4 (PMC4890528; doi:10.1186/s12866-016-0717-4)
Supplement: Additional file 2: — Information about the genes of C. pseudotuberculosis. All four genes were used in this work. These were united in the same alignment for phylogenetic analysis. (PDF 7 kb) [file 12866_2016_717_MOESM2_ESM.pdf]

Additional file 2. Information about the genes of *C. pseudotuberculosis*. All four genes were used in this work. These were united in the same alignment for phylogenetic analysis.

Gene Symbol	Gene Description	Biological process	Size
fusA	Translation elongation factors (GTPases)	Protein Biosynthesis	2127 bp
gapA	Glyceraldehyde-3-phosphate dehydrogenase	Glucose Metabolic Process	1005 bp
rpoB	DNA-directed RNA polymerase, beta subunit	Transcription, DNA-Templated	3537 bp
rsmE	16S ribosomal RNA methyltransferase	rRNA Processing	771 bp
