# Supplementary material for: Corynebacterium pseudotuberculosis may be under anagenesis and biovar Equi forms biovar Ovis: a phylogenic inference from sequence and structural analysis
Source: BMC Microbiol. 2016 Jun 2;16:100. doi: 10.1186/s12866-016-0717-4 (PMC4890528; doi:10.1186/s12866-016-0717-4)
Supplement: Additional file 3: — Ratio templates used for molecular modeling. € Resolution of the structure was determined by experimental methods of electron microscopy. (PDF 35 kb) [file 12866_2016_717_MOESM3_ESM.pdf]

Additional file 3. Ratio templates used for molecular modeling. € Resolution of the structure was determined by experimental methods of electron microscopy.

**Protein**	***fusA***		***gapA***		***rpoB***		***rsmE***	
**id PDB**	2XEX	3ZZ0	1HDG	3ZCX	1IW7	3IYD	4L69	1V6Z
**(chain)**	(A)	(A)	(O)	(A)	(C)	(C)	(A)	(A)
**Identity (%)**	59%	59%	43%	41%	49%	55%	47%	31%
**Coverage (%)**	99%	99%	70%	71%	95%	93%	92%	79%
**Resolution [Å]**	1.90	2.80	2.50	2.19	2.60	19.8€	2.90	2.00
