# Supplementary figures and images for: Corynebacterium pseudotuberculosis may be under anagenesis and biovar Equi forms biovar Ovis: a phylogenic inference from sequence and structural analysis
Source: BMC Microbiol. 2016 Jun 2;16:100. doi: 10.1186/s12866-016-0717-4 (PMC4890528; doi:10.1186/s12866-016-0717-4)

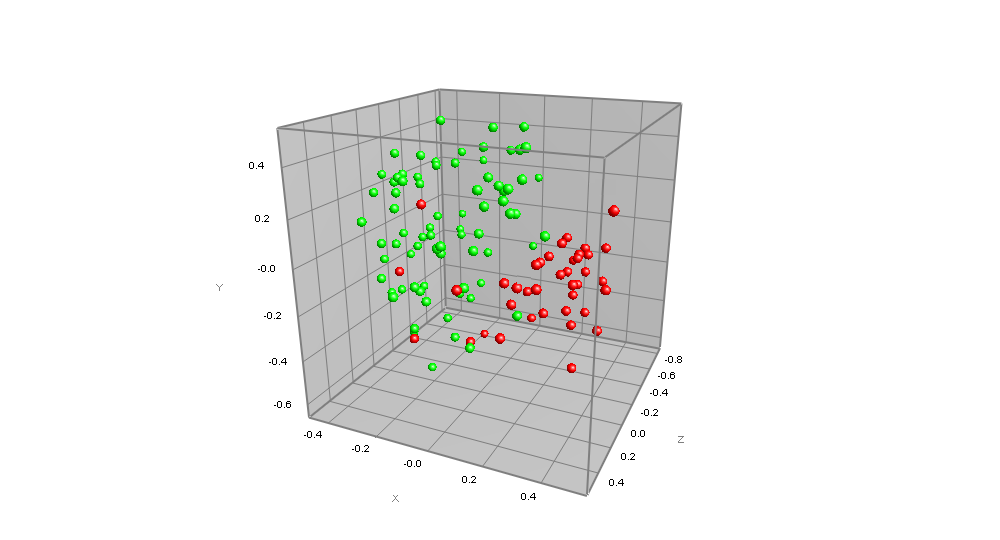

Supplement: Additional file 4: — Bionumerics 7.1 (Applied Maths, Sint-Martens-Latem, Belgium) (BMP 1582 kb) [file 12866_2016_717_MOESM4_ESM.bmp]

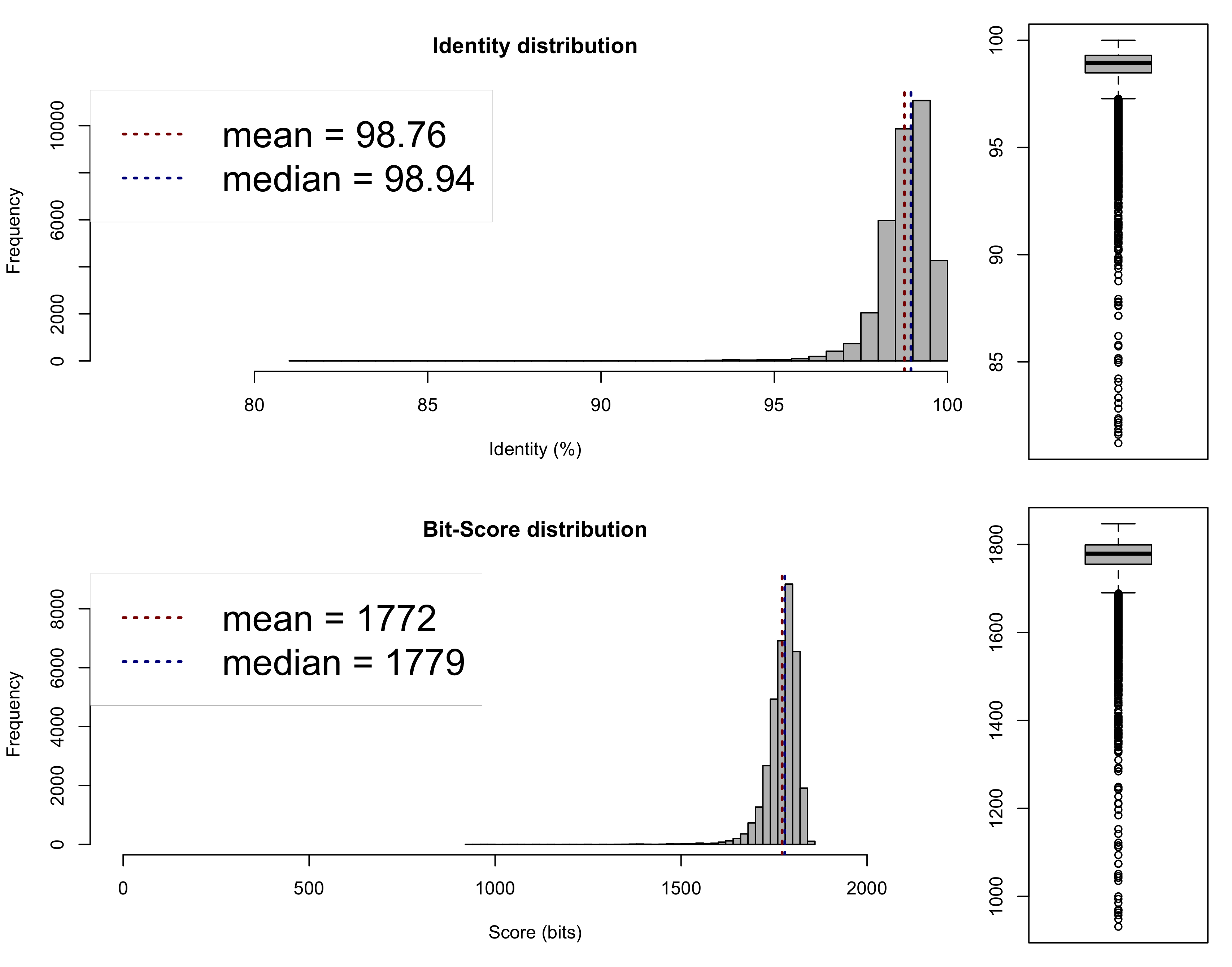

Supplement: Additional file 5: — Average Nucleotide Identity (ANI) from biovar ovis and biovar equi. Typically, the ANI values between genomes of the same species are above 95 %. Our results show the value 98.76 %, which we expected due to the results of phylogeny. One-way ANI 1: 98.64 % (SD: 1.15 %), from 67439 fragments. One-way ANI 2: 98.69 % (SD: 1.20 %), from 101821 fragments. Two-way ANI: 98.76 % (SD: 0.92 %), from 34975 fragments. (PNG 435 kb) [file 12866_2016_717_MOESM5_ESM.png]

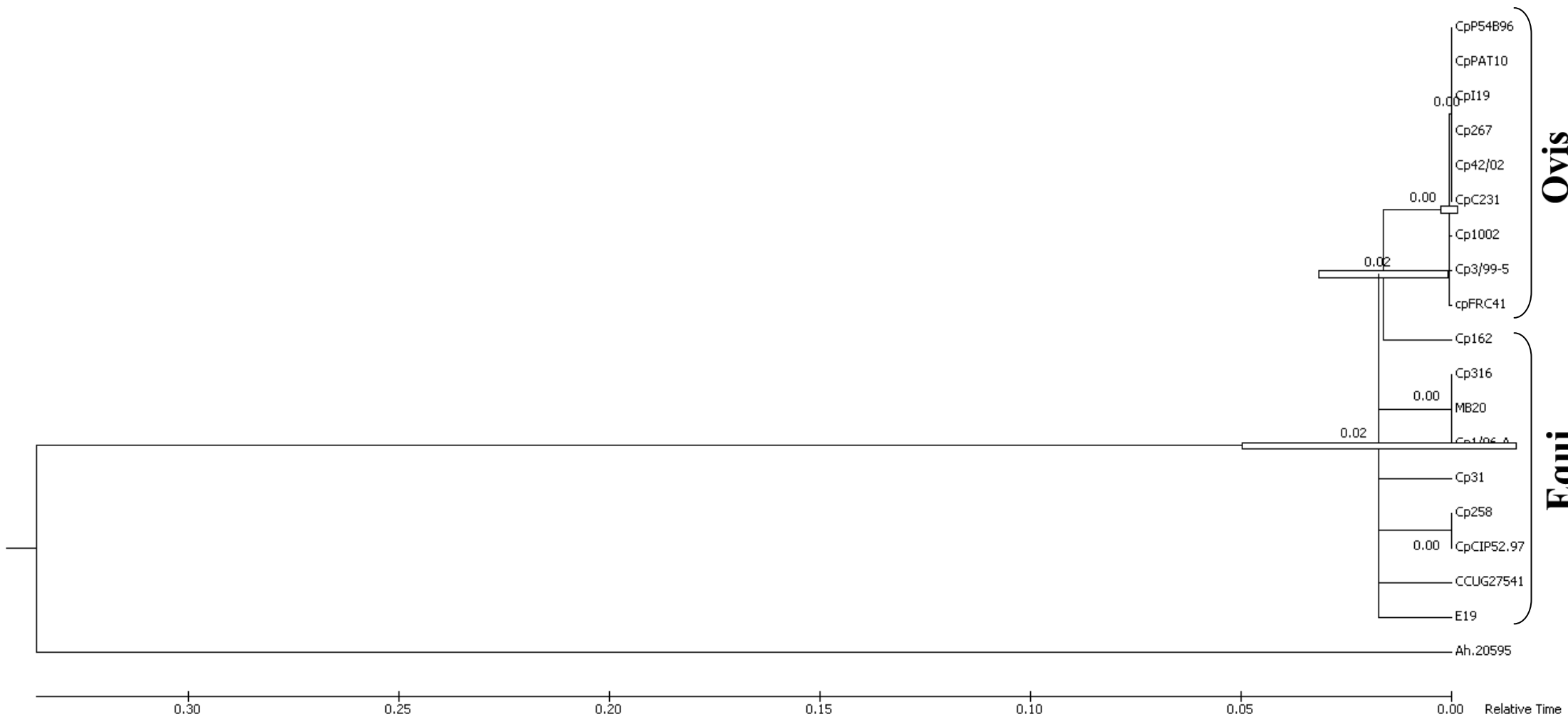

Supplement: Additional file 6: — Molecular clock analysis by Maximum Likelihood method. Relationship between molecular divergence and time. The analysis involved 19 amino acid sequences. All positions containing gaps and missing data were eliminated. There were a total of 6514 positions in the final dataset. Evolutionary analyses were conducted in MEGA6. (PDF 55 kb) [file 12866_2016_717_MOESM6_ESM.pdf]

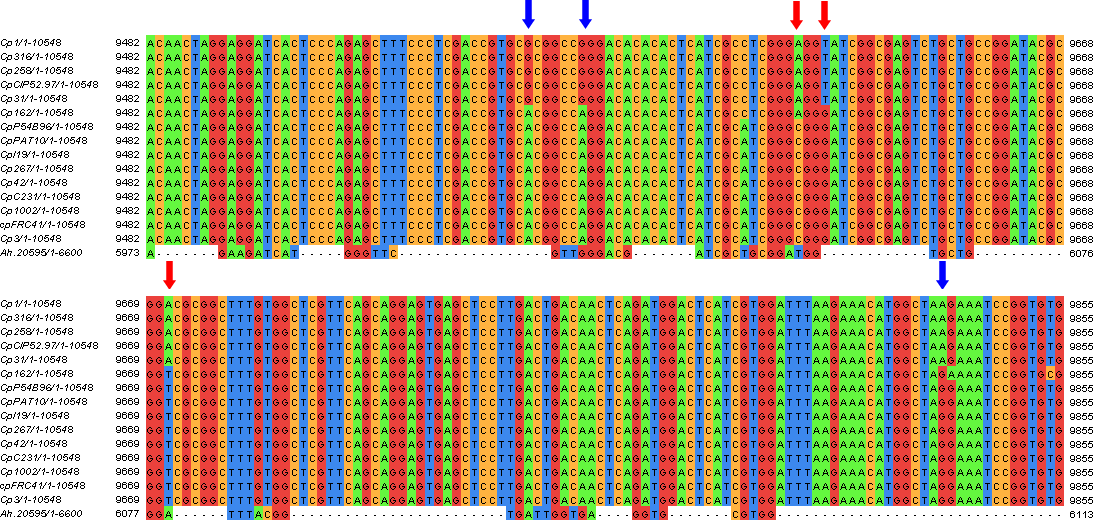

Supplement: Additional file 8: — Fragment of multiple sequence alignment. The figure shows transition (arrows in blue) and transversion (red arrow) point mutations observed in nucleotide sequences from both biovars Equi and Ovis. (TIF 164 kb) [file 12866_2016_717_MOESM8_ESM.tif]

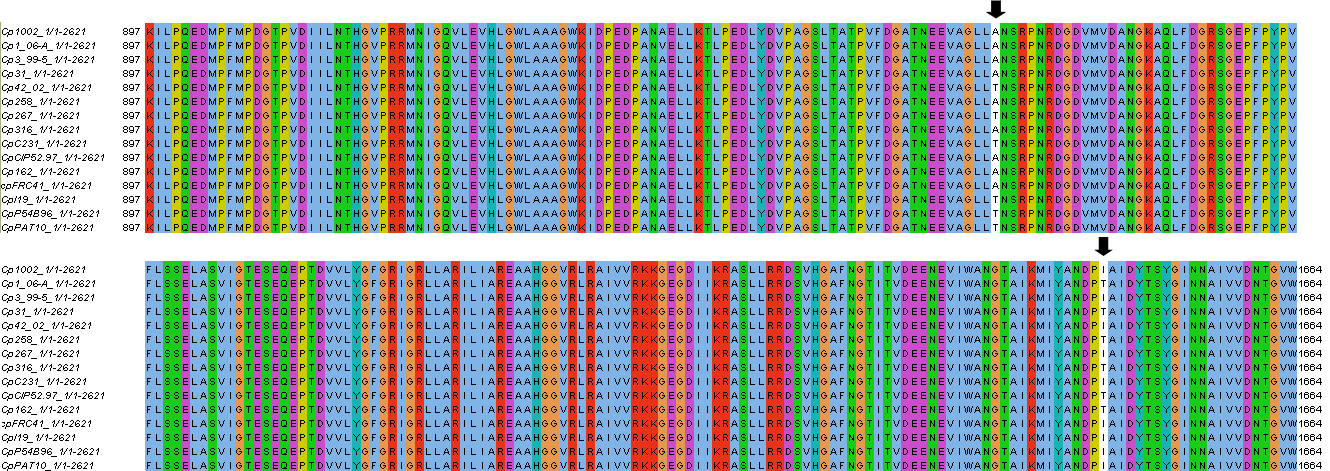

Supplement: Additional file 9: — Fragment of multiple sequence alignment of protein. The figure shows the substitutions of amino acids, in which most of the cases the changes modify the physicochemical characteristics. (TIF 216 kb) [file 12866_2016_717_MOESM9_ESM.tif]

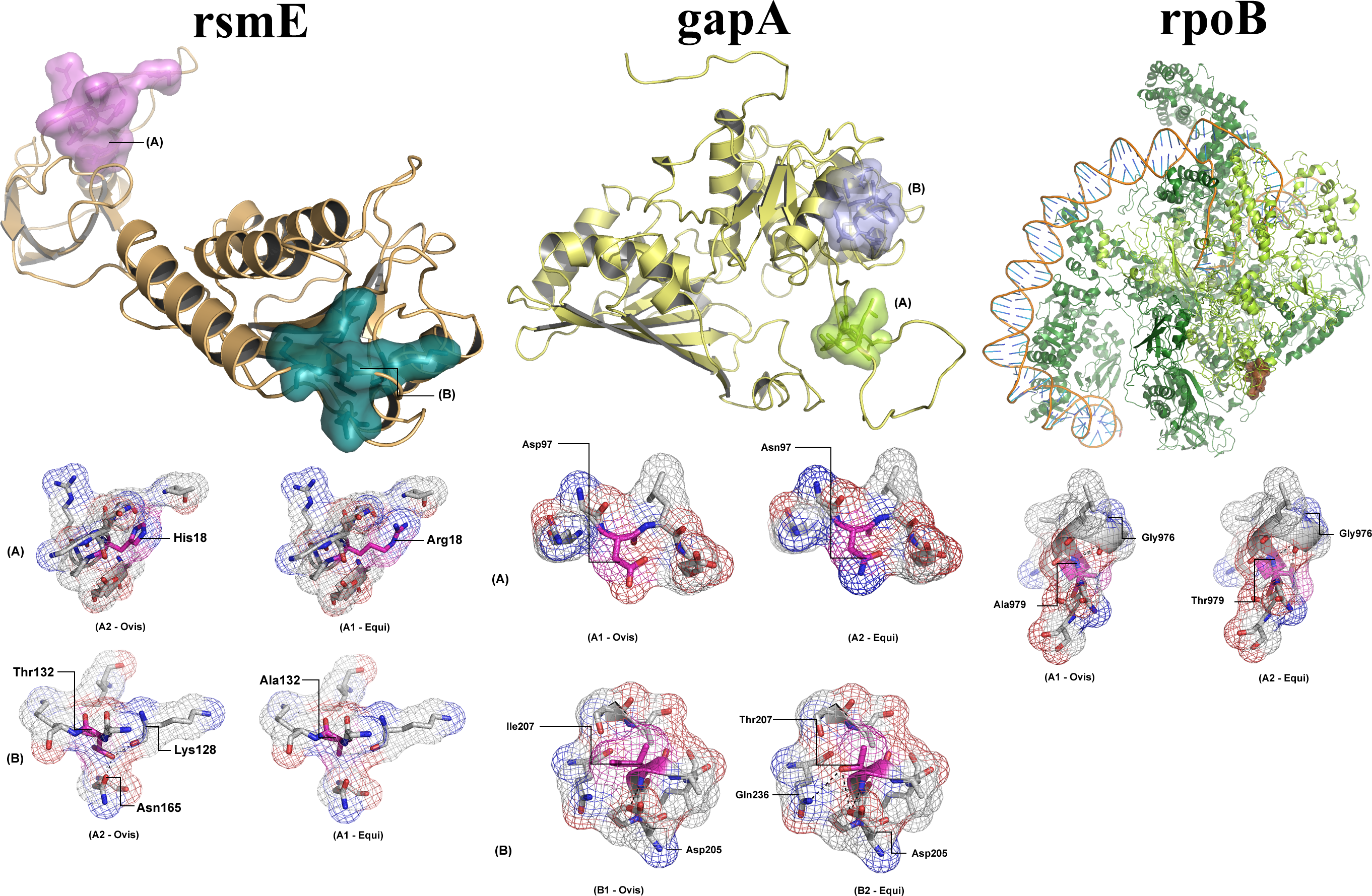

Supplement: Additional file 10: — Representation of the structure of gapA, rsmE and rpoB by molecular modeling. The molecular differences between the amino acid from biovars Equi and Ovis induce an increase in the number of chemical bonds between amino acids that are close to the variant residue. (TIF 4028 kb) [file 12866_2016_717_MOESM10_ESM.tif]

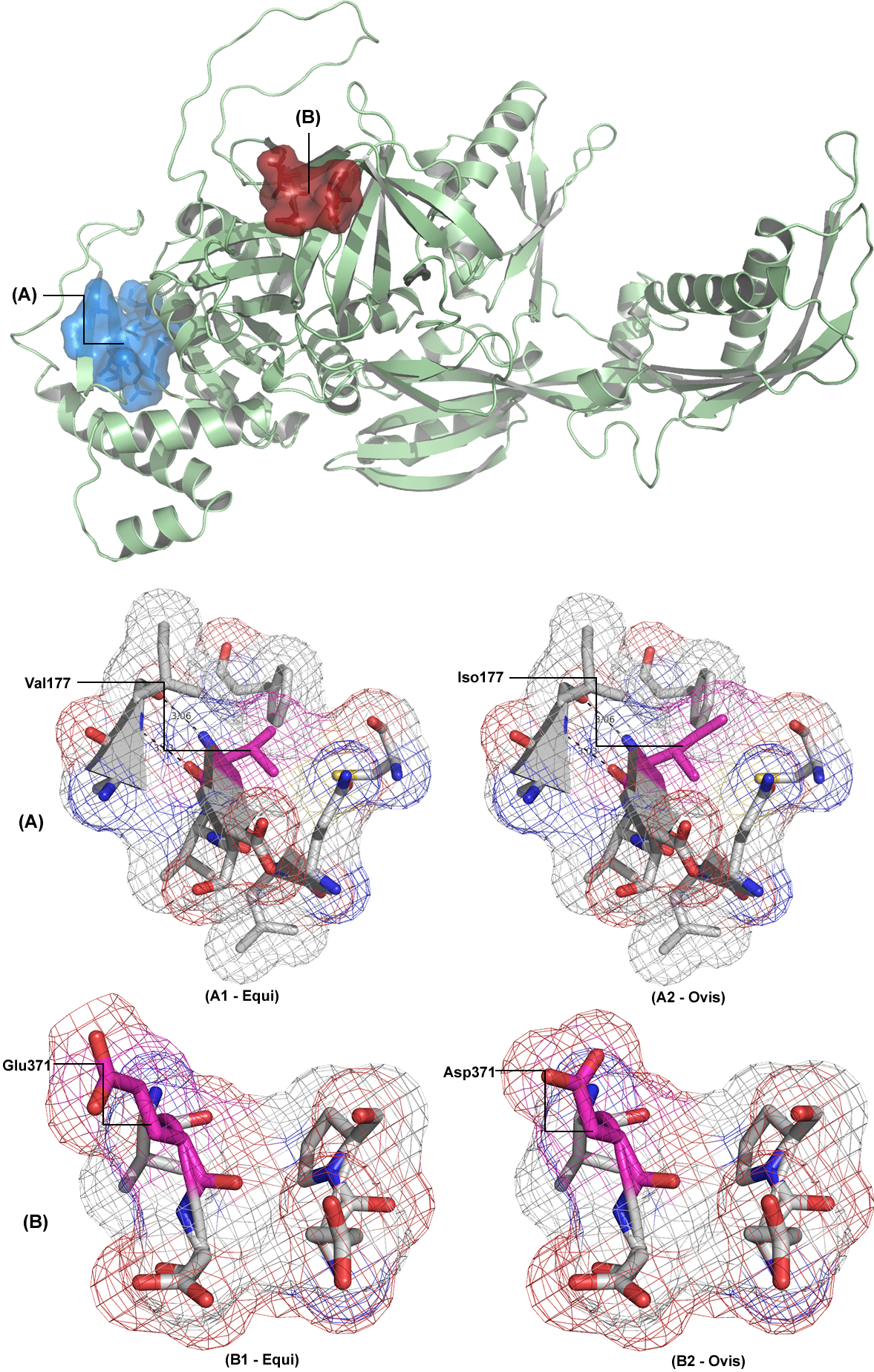

Supplement: Additional file 11: — Structure of the fusA protein in the biovars Equi and Ovis analyzed by molecular modeling [55]. Expansion of 4 angstroms (Å) from the variant amino acid where it is possible to identify clusters of neighboring residues interacting among themselves. (A) Variation between V ↔ I, (B) Variation between D ↔ E. (TIF 2499 kb) [file 12866_2016_717_MOESM11_ESM.tif]
