# Supplementary material for: Corynebacterium pseudotuberculosis may be under anagenesis and biovar Equi forms biovar Ovis: a phylogenic inference from sequence and structural analysis
Source: BMC Microbiol. 2016 Jun 2;16:100. doi: 10.1186/s12866-016-0717-4 (PMC4890528; doi:10.1186/s12866-016-0717-4)
Supplement: Additional file 7: — Results from comparative test with molecular clocks using the Maximum Likelihood method and without molecular clock of strains. (PDF 8 kb) [file 12866_2016_717_MOESM7_ESM.pdf]

Additional file 9. Results from comparative test with molecular clocks using the Maximum Likelihood method and without molecular clock of strains.

	**ln*L***	**Parameters**	**(+*G*))**	**(+*I*)**
**With Clock**	-9117104.656	19	n/a	n/a
**Without Clock**	-13870.885	32	n/a	n/a

NOTE. -- The molecular clock test was performed by comparing the ML value for the given topology with and without the molecular clock constraints under Tamura-Nei (1993) model [44]. The null hypothesis of equal evolutionary rate throughout the tree was rejected at a 5% significance level ($P = 0$). The analysis involved 15 nucleotide sequences. Codon positions included were 1st+2nd+3rd+Noncoding. All positions containing gaps and missing data were eliminated. There were a total of 7283 positions in the final dataset. Evolutionary analyses were conducted in MEGA6.
